# Modulating the Estrobolome and Inflammatory Microenvironment in Endometriosis: The Role of Microbiome-Targeted Interventions and Nutritional Compounds

**DOI:** 10.3390/nu18172883

**Published:** 2026-09-03

**Authors:** Stefania Greco, Giovanni Delli Carpini, Abel Duménigo Gonzàlez, Gaia Goteri, Andrea Ciavattini, Pasquapina Ciarmela

**Affiliations:** 1 Department of Life Sciences, Health and Health Professions, Link Campus University, 00165 Rome, Italy; s.greco@unilink.it; 2 Department of Clinical Sciences, Università Politecnica delle Marche, 60126 Ancona, Italy; g.dellicarpini@staff.univpm.it (G.D.C.); a.ciavattini@univpm.it (A.C.); 3 Department of Experimental and Clinical Medicine, Università Politecnica delle Marche, 60126 Ancona, Italy; a.dumenigo@pm.univpm.it; 4 Department of Biomedical Sciences and Public Health, Università Politecnica delle Marche, 60126 Ancona, Italy; g.goteri@staff.univpm.it

**Keywords:** endometriosis, estrobolome, gut microbiome, probiotics, oxidative stress, nutraceuticals, fertility, precision nutrition

## Abstract

Endometriosis is a chronic estrogen-dependent systemic inflammatory disorder characterized by ectopic implantation of endometrial-like tissue, primarily on the ovaries, pelvic peritoneum, and bowel. According to the World Health Organization (WHO) estimate updated in 2025, endometriosis affects approximately 10% (about 190 million) of reproductive-age women worldwide and is a major contributor to chronic pelvic pain, dysmenorrhea, dyspareunia, and infertility. Standard medical therapies focus on ovarian suppression, which alleviates symptoms but precludes conception and carries significant metabolic and skeletal adverse effects. Recent multi-omics research has raised interest in the gut microbiome and the estrobolome, defined as the microbial gene repertoire involved in estrogen metabolism, as potential modulators of systemic estrogen exposure and immune homeostasis. Altered microbiota composition and microbial β-glucuronidase activity may influence enterohepatic estrogen recirculation; however, endometriosis-specific evidence is predominantly associative or preclinical and does not establish a causal pathway. Dysbiosis and increased bacterial β-glucuronidase activity promote enterohepatic recirculation of estrogens, contributing to hyperestrogenism and ectopic lesion proliferation. Concurrently, oxidative stress, peritoneal inflammation, aberrant macrophage polarization, and neoangiogenesis sustain lesion survival and contribute to chronic pelvic pain. This comprehensive review synthesizes mechanistic, preclinical, and clinical evidence regarding microbiome-targeted interventions and nutritional compounds, including probiotics, prebiotics, N-acetyl cysteine (NAC), curcumin, resveratrol, epigallocatechin gallate (EGCG), omega-3 polyunsaturated fatty acids (PUFAs), and vitamin D, in modulating the estrobolome, immune responses, and oxidative microenvironment in endometriosis. We further discuss dietary patterns, bioavailability challenges, and the potential of precision nutrition to optimize reproductive outcomes. These approaches may be considered complementary or investigational adjuncts; current evidence is insufficient to demonstrate disease modification or improvements in spontaneous pregnancy, assisted reproductive technology (ART) outcomes, or live birth. Here, “fertility-sparing” denotes the absence of intentional ovulation suppression rather than proven fertility enhancement.

## 1. Introduction

**Figure 1:**
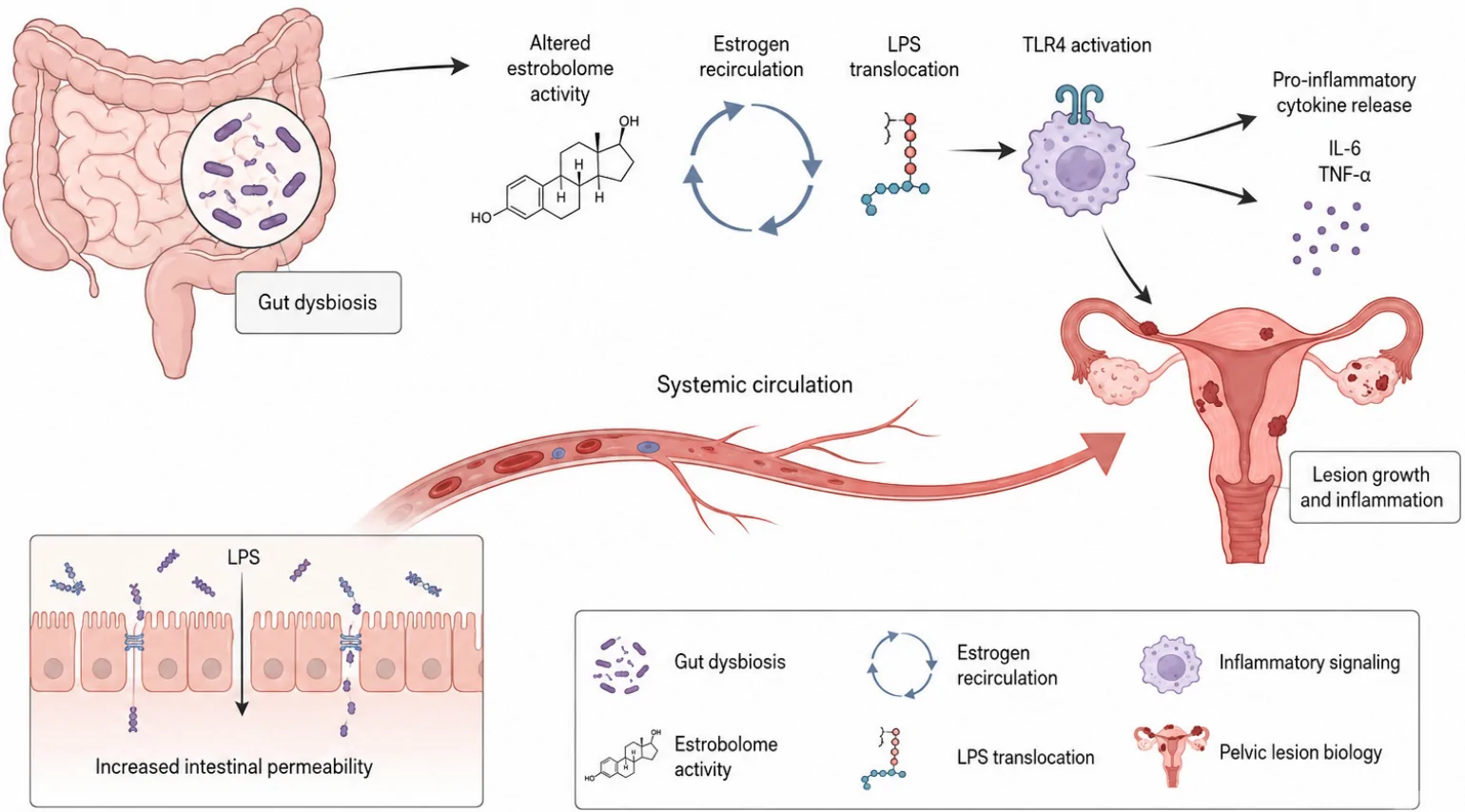
Anatomo-clinical gut–pelvic axis in endometriosis. The schematic links gut dysbiosis and estrobolome activity to estrogen recirculation, intestinal barrier dysfunction, LPS-related signaling, and pelvic lesion biology. The proposed pathways are schematic and do not imply established causality. The figure was created by the authors using both Canva and BioRender for the preparation of the graphical elements and images.

Endometriosis is a systemic, estrogen-dependent inflammatory disorder marked by the ectopic implantation and proliferation of endometrial-like glands and stroma outside the uterine cavity. Common implantation sites include the ovaries, pelvic peritoneum, and bowel, while extra-pelvic sites such as the lungs or brain are observed less frequently [1,2,3]. Epidemiological data estimate that 10–15% of women of reproductive age are affected, equating to over 190 million women globally [4,5,6]. However, due to the normalization of menstrual pain and the need for surgical or advanced imaging confirmation, the true prevalence is likely underestimated, with patients often facing a diagnostic delay of 7 to 10 years [7]. Clinical manifestations encompass a broad spectrum of debilitating symptoms, including chronic pelvic pain, severe dysmenorrhea, dyspareunia, and gastrointestinal or urinary disturbances [8]. Crucially, endometriosis is a leading cause of female infertility, affecting up to 50% of women struggling to conceive [9]. As a result, the disorder imposes a significant psychological, social, and economic burden, profoundly reducing patients’ quality of life and generating substantial healthcare costs [10,11]. The pathogenesis of endometriosis remains complex and multifactorial [12,13,14]. While Sampson’s theory of retrograde menstruation provides a foundational explanation for the anatomical displacement of endometrial cells into the peritoneal cavity [15], it is insufficient on its own. Retrograde menstruation occurs in up to 90% of menstruating women, yet only a fraction develops the disease. Other hypotheses, such as coelomic metaplasia and the stem cell theory, suggest alternative origins for ectopic lesions [16,17]. Regardless of the origin, the establishment and survival of ectopic tissue require additional permissive factors: immune dysfunction (such as altered macrophage polarization and reduced natural killercell cytotoxicity), impaired apoptosis, localized oxidative stress, and microbiome-mediated dysregulation [18,19,20]. At a molecular level, endometriotic lesions display progesterone resistance and increased estrogen receptor-beta (ER-β) expression [21,22,23]. This is coupled with the sustained activation of nuclear factor kappa B (NF-κB) and cyclooxygenase-2 (COX-2) upregulation, which drive enhanced prostaglandin synthesis, localized inflammation, and neoangiogenesis [24,25,26,27,28]. Current pharmacological treatments, such as combined oral contraceptives, progestins, and GnRH agonists or antagonists, primarily aim to suppress ovarian estrogen production to induce a hypoestrogenic state [22,29]. While effective for temporary symptom relief, these therapies present major clinical limitations: they do not address the underlying systemic inflammatory or endocrine drivers, are often poorly tolerated due to side effects (e.g., bone mineral density loss, mood alterations), and, most importantly, act as contraceptives [30]. This creates a therapeutic paradox for patients wishing to conceive, who are forced to discontinue medical therapy, leaving them exposed to disease progression and pain [31]. Consequently, there is a rapidly growing clinical interest in non-suppressive, adjunctive interventions targeting the gut-pelvic axis, immune modulation, and oxidative stress [20,32]. Recent multi-omics studies indicate that the gut microbiome, and specifically the estrobolome, the collection of microbial genes capable of metabolizing estrogens, plays a pivotal role in modulating systemic estrogen levels and immune homeostasis [33,34]. Dysbiosis, altered intestinal permeability (leaky gut), and systemic endotoxemia actively contribute to chronic inflammation, lesion persistence, and pain [35,36,37]. In this context, nutritional interventions and dietary supplements, including probiotics, prebiotics, polyphenols, omega-3 fatty acids, and vitamin D, emerge as promising strategies. These compounds may help restore microbial homeostasis, modulate immune responses, reduce oxidative stress, and indirectly regulate estrogen activity without suppressing ovulation [38,39,40,41]. This review aims to synthesize the current understanding of these nutritional interventions while distinguishing in vitro, animal, observational human, and interventional clinical evidence. By highlighting their mechanistic basis, preclinical evidence, and available clinical data, we discuss their potential role as complementary, non-ovulation-suppressive approaches within personalized reproductive care. A schematic overview of the proposed gut–pelvic pathways is provided in Figure 1.

## 2. Search Strategy and Selection Criteria

This narrative review was conducted through a literature search of PubMed, Scopus, Web of Science, and Google Scholar up to March 2026. The following keywords and Boolean combinations were used: “endometriosis” AND “gut microbiome”, “estrobolome”, “probiotics”, “prebiotics”, “short-chain fatty acids”, “oxidative stress”, “N-acetyl cysteine”, “curcumin”, “resveratrol”, “EGCG”, “omega-3 fatty acids”, “vitamin D”, “vitamin C”, “vitamin E”, “quercetin”, “alpha-lipoic acid”, “zinc”, “selenium”, “diet”, “fertility”, and “assisted reproductive technology”, “hormonal treatment”, and “menstrual cycle”. Priority was given to systematic reviews, meta-analyses, randomized controlled trials, prospective or observational clinical studies, and mechanistic preclinical studies directly addressing endometriosis or endometriosis-associated infertility. Evidence was categorized as in vitro, animal, observational human, or interventional human whenever feasible. Additional relevant articles were identified by screening the reference lists of selected publications. Studies not directly related to endometriosis, studies lacking mechanistic or clinical relevance, and non-English articles were excluded where appropriate. Because this is a narrative rather than systematic review, no formal risk-of-bias tool or quantitative meta-analysis was applied.

## 3. The Gut-Pelvic Axis and Estrobolome

### 3.1. Microbial Regulation of Estrogen Metabolism

The gut microbiome functions as a central regulator of systemic endocrine and immune processes. In estrogen-dependent pathologies, including endometriosis, the “estrobolome”, defined as the collective microbial genes encoding estrogen-metabolizing enzymes, including β-glucuronidases, may influence circulating estrogen exposure [35,42,43]. Importantly, microbiota composition and estrobolome function are not synonymous: taxonomic abundance does not directly measure microbial gene expression or enzyme activity.

Under physiological conditions, estrogens are conjugated in the liver with glucuronic acid or sulfate, rendering them water-soluble for biliary excretion and elimination via feces [44,45]. However, in the intestinal tract, multiple bacterial groups, including members of the *Clostridium*, *Bacteroides*, and *Ruminococcus* genera, can encode β-glucuronidases [46,47]. This enzyme deconjugates the excreted estrogens back into their active, lipophilic forms, allowing them to be reabsorbed through the intestinal mucosa into the systemic circulation [48]. Enzyme activity is gene- and strain-dependent and cannot be inferred reliably from genus abundance alone [46,47].

In endometriosis, altered microbiota composition and β-glucuronidase-related activity have been proposed as potential modifiers of enterohepatic estrogen recirculation [36]. However, direct endometriosis-specific evidence linking microbial β-glucuronidase activity to circulating estrogen levels and lesion progression remains limited, and current data do not establish causality. Some human cohorts report differences in *Lactobacillus* spp. and other microbial taxa [20,49], but these studies are mostly cross-sectional and heterogeneous. Consequently, microbiome- or diet-mediated modulation of estrobolome function should currently be considered a hypothesis-generating research strategy rather than an established method for lowering systemic estrogen burden [3,50,51,52]. Microbial changes may precede disease, arise secondary to inflammation, diet, bowel symptoms, surgery, or hormonal treatment, or participate bidirectionally.

### 3.2. Intestinal Permeability and LPS-Induced Endotoxemia

Beyond endocrine modulation, gut dysbiosis profoundly impacts the local immune landscape by compromising the intestinal epithelial barrier. An imbalance in the gut microbiota can lead to the downregulation of tight junction proteins (such as zonulin and occludin), resulting in increased intestinal permeability, commonly referred to as “leaky gut” [53,54]. This compromised barrier allows lipopolysaccharides (LPS) derived from the cell walls of Gram-negative bacteria, along with other microbial metabolites, to translocate from the gut lumen into the systemic circulation and the peritoneal cavity. Once in the pelvic microenvironment, LPS acts as a potent endotoxin. It binds to toll-like receptor 4 (TLR4) on circulating monocytes and resident peritoneal macrophages, triggering the activation of the NF-κB signaling pathway [55,56,57]. This continuous TLR4-NF-κB activation shifts macrophages toward a pro-inflammatory state, leading to the massive release of cytokines and chemokines, including interleukin (IL)-1beta, IL-6, IL-8, and tumor necrosis factor-alpha (TNF-α) [58]. This cytokine storm not only promotes the survival and adherence of retrograde endometrial cells but also drives localized fibrosis, neoangiogenesis, and neurogenesis, which are directly responsible for chronic pelvic pain. Elevated levels of serum and peritoneal LPS correlate strongly with the severity of endometriosis and pelvic pain, highlighting the gut-pelvic inflammatory axis as a critical therapeutic target [59]. Dietary patterns directly influence this intestinal permeability. High-fiber diets support tight junction integrity by promoting the production of short-chain fatty acids (SCFAs) and enhancing microbial diversity. Conversely, Western-style diets, rich in ultra-processed foods, saturated fats, and refined carbohydrates, have been associated with less favorable microbial and inflammatory profiles, although endometriosis-specific causal evidence remains limited [60,61,62]. These proposed connections are summarized in Figure 1.

## 4. Vaginal Microbiome and Reproductive Immunity

While the gut microbiome regulates systemic inflammation and circulating estrogens, the local microbiomes of the lower and upper female reproductive tracts are equally vital for maintaining pelvic immune homeostasis and fertility. In healthy individuals, *Lactobacillus*-dominant vaginal communities are common; frequently described dominant species include *L. crispatus*, *L. gasseri*, *L. iners*, and *L. jensenii* [63,64,65]. Species and strains within the same genus can have distinct biological properties. In women with endometriosis, however, the reproductive microbiome is often significantly altered. Studies report reduced Lactobacillus abundance and increased microbial diversity in both the vaginal and cervical tracts, with an increased relative abundance of potentially pathogenic bacteria (such as *Gardnerella*, *Prevotella*, and *Atopobium*) [66,67]. This local dysbiosis is associated with elevated cervicovaginal inflammation, altered cytokine profiles, and impaired mucosal immunity. Crucially, emerging evidence indicates that this dysbiotic environment is not confined to the vagina but can ascend to the upper reproductive tract, altering the endometrial microbiome. An inflamed, non-*Lactobacillus*-dominant endometrial cavity negatively impacts endometrial receptivity, impairing decidualization and embryo implantation. Associations between non-*Lactobacillus*-dominant reproductive-tract communities and implantation or IVF outcomes are biologically plausible, but endometriosis-specific evidence remains limited and largely observational. Furthermore, crosstalk between the gut and vaginal microbiomes, mediated by systemic immune signaling and the migration of immune cells, influences macrophage polarization and natural killer NK) cell activity in the peritoneal fluid. This complex gut-pelvic-immune axis further supports the integration of microbiome-targeted therapies not only to manage pain but also to optimize the reproductive microenvironment for conception [20,36]. Table 1 summarizes representative taxa and their proposed relevance while separating taxonomic associations from functional estrobolome evidence.

## 5. Oxidative Stress and Iron Overload

Within the gut–pelvic framework, local iron-driven oxidative stress and microbiome-related inflammatory signaling should be viewed as potentially convergent rather than as a single causal pathway. Retrograde menstrual blood can increase heme/iron and Fenton chemistry in the peritoneal cavity, whereas microbial products such as LPS may activate TLR4/NF-κB signaling [56,57,58,68,69,70].

These mechanisms can converge on cytokine production and reactive oxygen species, but current evidence does not demonstrate that pelvic iron overload is caused by gut dysbiosis.

The peritoneal microenvironment in women with endometriosis is profoundly altered, characterized by a state of chronic oxidative stress and iron toxicity [19,57,58]. According to the retrograde menstruation theory, the reflux of menstrual blood introduces erythrocytes and endometrial cellular debris into the pelvic cavity. The subsequent hemolysis of these erythrocytes leads to an accumulation of free iron, which catalyzes the generation of highly toxic hydroxyl radicals via the Fenton reaction [69,70].

This iron overload and the resulting overproduction of reactive oxygen species (ROS) have multiple detrimental effects. First, excessive ROS induces lipid peroxidation, DNA damage, and protein degradation within the pelvic microenvironment [58,71]. Recently, this iron-dependent accumulation of lipid peroxides has been linked to ferroptosis, a unique form of programmed cell death. However, endometriotic cells paradoxically develop resistance to ferroptosis, allowing ectopic lesions to survive and proliferate despite the toxic environment [72].

Second, the chronic oxidative stress heavily influences local immune responses. Resident peritoneal macrophages, exposed to continuous iron overload, undergo polarization toward a pro-fibrotic and tissue-remodeling M2 phenotype. These M2 macrophages secrete elevated levels of transforming growth factor-beta (TGF-β) and vascular endothelial growth factor (VEGF), actively promoting fibrogenesis, neuroangiogenesis, and lesion adherence [68,73,74].

**Figure 2:**
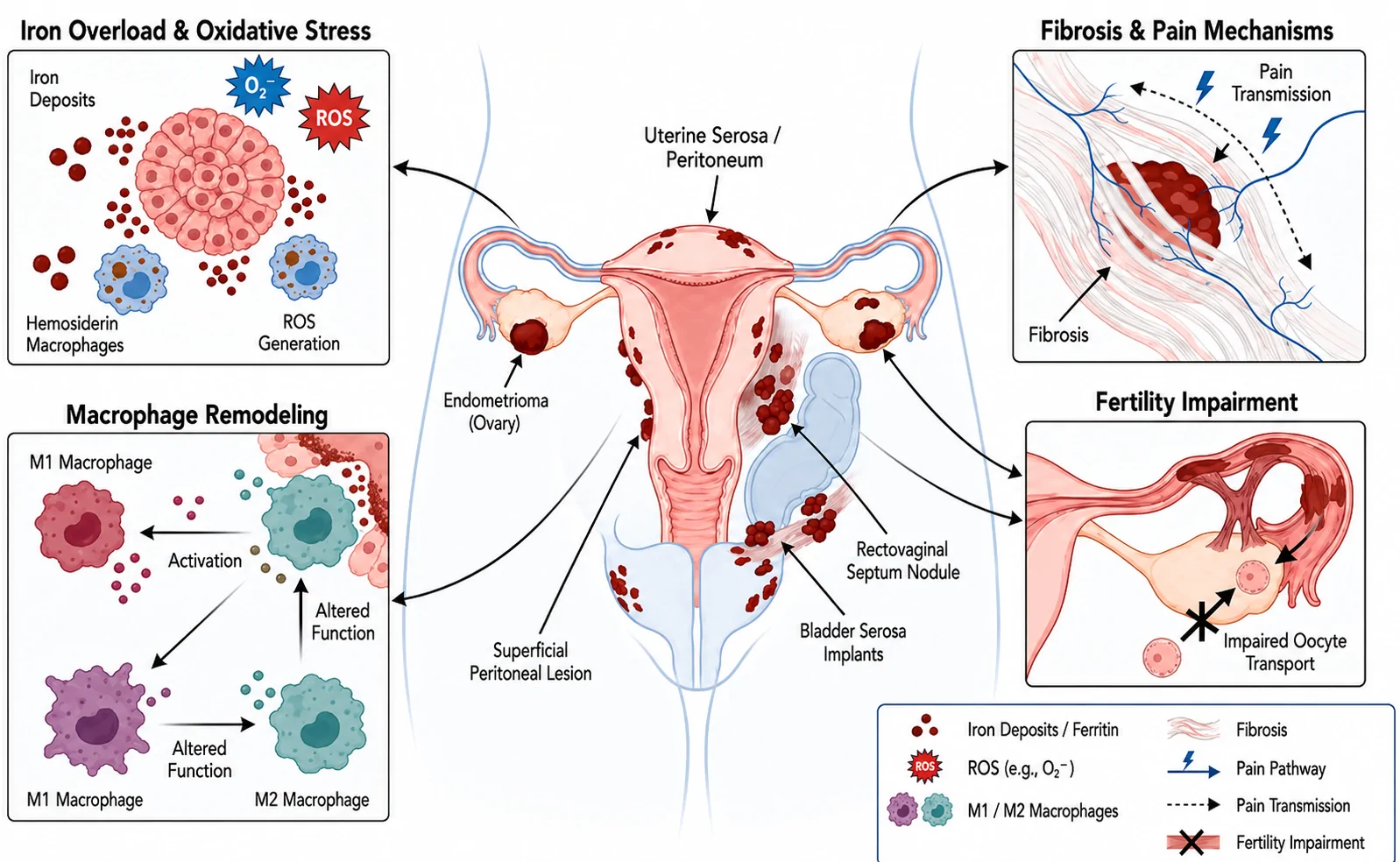
Anatomo-clinical pelvic microenvironment in endometriosis. Common lesion sites are shown together with iron overload, ROS generation, macrophage remodeling, fibrosis, pain, and fertility-related impairment. The proposed pathways are schematic and do not imply established causality. The figure was created by the authors using both Canva and BioRender for the preparation of the graphical elements and images.

For reproductive health, oxidative stress has been associated with impaired follicular and oocyte environments in endometriosis, although links to embryo competence and live birth are not uniform across studies [19,75]. Antioxidant interventions are therefore biologically plausible, but their clinical effects must be judged separately from mechanistic evidence. The Atlas of Stress Response Activity (ASTRA) integrates transcriptomic responses of human cell lines to stressors, including H_2_O_2_, and may support future hypothesis generation for oxidative-stress pathways; however, it is not endometriosis-specific and requires disease-specific validation [76]. Figure 2 summarizes the principal pelvic pathways.

## 6. Microbiome-Targeted Interventions: Experimental and Early Clinical Evidence

### 6.1. Probiotics

Given the central role of dysbiosis in the gut–pelvic axis, the administration of live beneficial microorganisms, namely probiotics, represents a biologically plausible intervention. Supplementation with specific, well-characterized strains, primarily belonging to the *Lactobacillus* and *Bifidobacterium* genera, has been shown to modulate systemic estrogen levels and immune responses effectively [41,77,78]. Mechanistically, selected probiotic strains may alter microbial competition and β-glucuronidase-related activity. Whether these effects meaningfully change enterohepatic estrogen recirculation or circulating estrogen exposure in women with endometriosis remains unproven [79]. Selected probiotics also show immunomodulatory effects in preclinical studies. Preclinical studies support immune effects of selected *Lactobacillus* preparations, but these animal/mechanistic findings should be separated from human clinical evidence [41].

Human clinical evidence is preliminary. A randomized double-blind placebo-controlled study evaluated heat-killed *L. gasseri* OLL2809 at 100 mg/day for 12 weeks and reported improvement in menstrual pain and dysmenorrhea [41]. A separate small placebo-controlled trial used one LactoFem^®^ capsule/day for 8 weeks, containing 10^9^ total colonies of *L. acidophilus*, *L. plantarum*, *L. fermentum*, and *L. gasseri*, and reported short-term pain improvement [77]. These studies do not establish a clinically validated probiotic regimen; no consensus exists regarding optimal strain, viable versus heat-killed preparation, colony-forming-unit dose, duration, route, or reproductive benefit.

### 6.2. Prebiotics and Short-Chain Fatty Acids

Prebiotics, such as inulin, fructooligosaccharides (FOS), and galactooligosaccharides (GOS), are non-digestible fibers that serve as specific substrates for beneficial gut microbiota. The bacterial fermentation of these fibers yields short-chain fatty acids (SCFAs), primarily acetate, propionate, and butyrate, which are crucial mediators of gut-immune cross-talk [80,81]. Butyrate, in particular, exhibits profound anti-inflammatory and epigenetic properties. It functions as a natural histone deacetylase (HDAC) inhibitor. By promoting histone acetylation, butyrate alters gene expression in ectopic endometrial cells, downregulating proliferative pathways and inducing apoptosis [82]. Additionally, SCFAs are the primary energy source for colonocytes and are essential for maintaining intestinal barrier integrity. They upregulate the expression of tight junction proteins (like zonulin and occludin), directly counteracting the “leaky gut” phenomenon, thereby preventing LPS translocation and the subsequent endotoxemia that drives pelvic inflammation [83,84]. Thus, prebiotics may support microbial metabolism and intestinal barrier integrity, but the pathway from these mechanisms to clinical improvement in endometriosis remains unproven. No endometriosis-specific clinical trial currently defines an effective inulin, FOS, or GOS dose or treatment duration.

## 7. Dietary Supplements and Natural Compounds

### 7.1. N-Acetyl Cysteine (NAC)

N-Acetyl Cysteine (NAC) is a well-tolerated synthetic derivative of the endogenous amino acid L-cysteine and serves as a direct precursor to glutathione (GSH), the body’s primary intracellular antioxidant. In the context of endometriosis, where iron overload and ROS production drive disease progression, NAC administration effectively replenishes the GSH pool, neutralizing free radicals and restoring the cellular redox balance [85,86,87]. Beyond its antioxidant properties, NAC exerts potent anti-proliferative effects. Mechanistically, it suppresses the PI3K/AKT/mTOR signaling pathway and downregulates the expression of inflammatory cytokines in endometriotic stromal cells, promoting a switch from proliferation to apoptosis. Clinically, NAC is among the better-studied nutraceuticals in endometriosis, although the clinical evidence remains limited. An observational study reported that oral supplementation with NAC (600 mg, three times daily, for three consecutive days per week) over three months led to a significant reduction in the mean diameter of ovarian endometriomas, preventing scheduled surgeries in over half of the treated patients. This intermittent schedule should be described as the protocol studied rather than as a validated dosing strategy [86]. Although NAC is among the most clinically investigated nutraceuticals in endometriosis, available studies remain limited by design, sample size, and heterogeneity of endpoints. Effects on spontaneous pregnancy or live birth have not been established.

### 7.2. Curcumin

Curcumin, the primary bioactive polyphenol derived from *Curcuma longa*, is widely recognized for its potent anti-inflammatory and anti-angiogenic properties [88]. In endometriotic models, curcumin modulates inflammatory signaling by inhibiting IKB phosphorylation, thereby preventing NF-κB nuclear translocation. This blockade significantly downregulates the downstream transcription of cyclooxygenase-2 (COX-2) and reduces the synthesis of prostaglandin E2 (PGE2), which are primary mediators of pelvic pain and dysmenorrhea. Furthermore, curcumin reduces the expression of matrix metalloproteinases (MMPs), impeding the invasion of ectopic cells [89]. Despite these promising mechanisms, the clinical utility of unformulated curcumin is severely limited by its poor aqueous solubility, rapid hepatic glucuronidation, and low systemic bioavailability. Formulation strongly affects curcumin exposure; however, improved bioavailability should not be assumed to translate into clinical efficacy in endometriosis. Despite promising mechanistic data, endometriosis-specific clinical evidence for curcumin remains limited, and its therapeutic potential depends strongly on formulation and bioavailability. A placebo-controlled trial of curcumin 500 mg twice daily for 8 weeks did not show clear superiority for painful symptoms [90], whereas a later nanocurcumin study used adjunctive dienogest, limiting attribution to curcumin alone [91].

### 7.3. Resveratrol

Resveratrol (3,5,4′-trihydroxystilbene) is a natural polyphenol found abundantly in the skin of red grapes and berries. Structurally resembling diethylstilbestrol, resveratrol functions as a selective estrogen receptor modulator (SERM). Depending on the specific tissue and local estrogen concentration, it can competitively bind to estrogen receptors (ERs), antagonizing the proliferative effects of systemic hyperestrogenism on ectopic lesions. Additionally, resveratrol acts as a potent activator of SIRT1 (Sirtuin 1), an NAD+-dependent deacetylase. SIRT1 activation by resveratrol downregulates inflammatory pathways, reduces ROS production, and inhibits the expression of MMP-2 and MMP-9, thereby restricting the invasive capacity and angiogenesis of endometriotic tissue. Preclinical murine models have consistently shown that resveratrol administration significantly reduces the volume and vascularization of endometriotic implants [92,93]. Clinical findings remain mixed, particularly because some studies evaluated resveratrol as an adjunct to hormonal therapy, limiting its interpretation as a fertility-sparing intervention. Human findings remain limited and inconsistent, particularly because several studies used resveratrol together with hormonal treatment [94,95]; independent efficacy and fertility benefits are unproven.

### 7.4. Epigallocatechin Gallate (EGCG)

Epigallocatechin gallate (EGCG), the most abundant and active catechin in green tea (*Camellia sinensis*), exhibits profound anti-angiogenic and anti-fibrotic properties [96,97]. Endometriotic lesions are highly dependent on the formation of a new vascular network for their survival and expansion. EGCG potently disrupts this process by inhibiting the expression of VEGF and hypoxia-inducible factor 1-alpha (HIF-1alpha). This rationale is supported by immunohistochemical evidence showing VEGF expression in ovarian endometriomata and by studies demonstrating the involvement of proangiogenic molecules, HIF-1alpha, and nitric oxide synthase isoforms in ovarian endometriotic cysts [98,99]. By starving the ectopic lesions of their blood supply, EGCG prevents the progression of early implants. Furthermore, recent studies highlight its role in modulating fibrosis, a major cause of chronic pelvic pain and anatomical distortion in deep infiltrating endometriosis, by suppressing the TGF-β signaling pathway and reducing collagen deposition in endometriotic stromal cells [100,101]. At present, EGCG evidence in endometriosis remains largely experimental, and clinical trials are needed to determine effective dosing, safety, and reproductive outcomes. Accordingly, EGCG should be regarded as a preclinical candidate rather than a clinically validated endometriosis therapy. In the SAGE randomized placebo-controlled trial, fish-oil supplementation did not significantly reduce pain compared with placebo in adolescents and young women [39]; standardized therapeutic dosing and reproductive benefits remain unproven.

### 7.5. Omega-3 Polyunsaturated Fatty Acids (EPA/DHA)

The modern Western diet is characteristically skewed towards a high Omega-6 to Omega-3 ratio, providing an abundance of arachidonic acid (AA), which fuels the synthesis of pro-inflammatory prostaglandins (PGE2) and leukotrienes [102]. Supplementation with omega-3 polyunsaturated fatty acids (PUFAs), specifically eicosapentaenoic acid (EPA) and docosahexaenoic acid (DHA), directly counters this inflammatory cascade. EPA and DHA competitively inhibit the COX and LOX enzymatic pathways, reducing the production of pain-inducing prostaglandins. Crucially, EPA and DHA are not merely anti-inflammatory; they serve as the immediate precursors for specialized pro-resolving mediators (SPMs), including resolvins, protectins, and maresins. These bioactive lipid mediators actively resolve chronic inflammation, promote the clearance of cellular debris by macrophages, and restore tissue homeostasis, offering a dual mechanism for pain relief and pelvic immune regulation [103]. Clinical studies suggest potential anti-inflammatory and analgesic effects, but differences in dose, EPA/DHA ratio, treatment duration, and endpoints limit definitive conclusions.

### 7.6. Vitamin D

Vitamin D, acting via its active metabolite calcitriol (1,25-dihydroxyvitamin D3), behaves as a steroid hormone with extensive immunomodulatory and endocrine functions. The vitamin D receptor (VDR) is widely expressed in both normal endometrium and endometriotic lesions [104]. Experimental studies describe vitamin D-related immunomodulatory effects, including modulation of pro-inflammatory cytokines and regulatory T-cell pathways. Experimental literature also supports a role for vitamin D signaling in epithelial barrier biology; however, these mechanisms have not established an endometriosis-specific reduction in intestinal permeability, LPS translocation, or systemic endotoxemia. Observational studies frequently report a high prevalence of Vitamin D deficiency in women with endometriosis, correlating with disease severity and the magnitude of pelvic pain. Vitamin D supplementation may be most relevant in deficient patients, but its disease-modifying role in endometriosis remains uncertain.

### 7.7. Additional Antioxidant and Micronutrient Compounds

Quercetin has antioxidant and anti-inflammatory properties. Rat models of experimental endometriosis have reported reductions in lesion-related or inflammatory parameters [105], but endometriosis-specific human randomized trials are lacking; efficacy, dose, and reproductive safety cannot be inferred from preclinical data. alpha-lipoic acid (ALA) is a redox-active cofactor. In the LEAP open-label study, a combination of NAC, ALA, and bromelain for six months was associated with reduced pelvic pain and analgesic use [106]. Because ALA was combined with other active ingredients and there was no placebo comparator, its independent effect and a validated monotherapy dose remain unknown. Vitamins C and E have small randomized human studies in endometriosis. Trials using vitamin C 1000 mg/day with vitamin E 800–1200 IU/day for 8 weeks reported reductions in selected oxidative-stress markers and pain [107,108]. These data do not establish lesion regression, pregnancy, or live-birth benefit, and optimal long-term dosing remains undefined. Zinc and selenium contribute to antioxidant and immune pathways. Zinc evidence in endometriosis is observational and insufficient to support routine supplementation without documented deficiency [109]. A 2026 triple-blind trial evaluated selenium 200 μg/day for three months as an adjunct to dienogest and reported greater reductions in endometrioma size and pain than placebo plus dienogest [110]; because both groups received hormonal therapy, this is adjunctive rather than standalone evidence.

## 8. Dietary Patterns and Lifestyle Modifications

Beyond isolated supplements, overall dietary patterns exert a profound, continuous influence on gut microbiome composition, systemic estrogen metabolism, and the inflammatory tone of the pelvic microenvironment [111]. The Mediterranean diet, characterized by a high intake of fruits, vegetables, whole grains, legumes, nuts, and extra-virgin olive oil, is consistently associated with a reduced risk of endometriosis and improved symptom management. This protective effect is mediated by the synergistic action of abundant dietary polyphenols, a high fiber content, and a favorable omega-3 to omega-6 fatty acid ratio. High dietary fiber specifically supports the growth of SCFA-producing bacteria, strengthens the intestinal epithelial barrier, and promotes the fecal excretion of estrogen metabolites, thereby limiting their enterohepatic recirculation [112]. Furthermore, dietary phytoestrogens, such as soy isoflavones and flaxseed lignans, exhibit a weak affinity for estrogen receptors, potentially exerting a competitive, modulatory effect in an estrogen-dominant environment [113]. Conversely, Western-style diets, dense in ultra-processed foods, saturated and trans fats, refined carbohydrates, and red meat, are powerful drivers of dysbiosis and intestinal permeability. This dietary pattern fosters the proliferation of pro-inflammatory, LPS-producing bacteria, directly fueling the systemic endotoxemia that sustains endometriotic lesions and pelvic pain. Additionally, the high consumption of ultra-processed foods often correlates with increased exposure to environmental endocrine-disrupting chemicals (EDCs), such as bisphenol A (BPA) and phthalates used in food packaging. These exposures may interfere with hormonal homeostasis and have been investigated in relation to endometriosis [114], but direct effects on the estrobolome and disease progression remain uncertain. Lifestyle co-exposures—including physical activity, sleep, smoking, alcohol, bowel symptoms, and stress—may also influence inflammation, symptom perception, dietary behavior, and microbiome composition, but intervention data specific to endometriosis are sparse. Restrictive elimination diets should not be recommended routinely without a symptom-based indication or nutritional supervision. Dietary counseling should distinguish improvements in diet quality or gastrointestinal symptoms from unproven claims of lesion regression, disease modification, or enhanced fertility.

## 9. Clinical Translation: Challenges and Considerations

While the mechanistic rationale for nutritional and microbiome-targeted interventions in endometriosis is compelling, several critical challenges must be addressed to facilitate their robust clinical translation.

First, the inherent interindividual variability of the human microbiome means that a generalized approach to probiotics, prebiotics, and diets is often inadequate. Potential sources of variability include age, body mass index, geography/ethnicity, habitual diet, gastrointestinal symptoms, smoking/alcohol, recent antibiotics or probiotics, hormonal therapy, menstrual cycle phase, pregnancy history, endometriosis phenotype/stage, prior surgery, and baseline nutrient status [20,36,47,64,115,116,117]. Technical factors including sampling site/timing, storage, DNA extraction, sequencing platform, taxonomic database, and batch effects can further alter apparent microbial signatures. A taxonomic profile does not itself quantify estrobolome activity; functional assays of microbial genes, β-glucuronidase activity, and estrogen metabolites are needed.

Second, the poor pharmacokinetic profiles of many promising natural compounds remain a significant hurdle. As highlighted previously, potent polyphenols like curcumin, resveratrol, and EGCG suffer from low aqueous solubility, rapid hepatic glucuronidation, and consequently, limited systemic exposure. The future of nutraceutical therapy in endometriosis relies heavily on the widespread adoption of advanced delivery systems, such as liposomal encapsulation, phytosome complexes, and nanoparticle-based carriers, to achieve clinically relevant tissue concentrations at the ectopic lesion sites [118].

Third, most existing clinical evidence is derived from small, heterogeneous, or observational studies with variable follow-up periods. To establish these interventions within standard gynecological guidelines, large-scale, multi-center, randomized, placebo-controlled trials using standardized formulations are urgently required [119].

From a clinical perspective, it is important to emphasize that, given the current state of evidence, nutritional and microbiome-targeted interventions cannot be considered disease-modifying therapies but rather complementary strategies to standard endometriosis management [120]. They may represent an option for patients seeking pregnancy who are not eligible for hormonal therapy or for women who experience persistent symptoms despite conventional treatment or who cannot tolerate hormonal therapy [120]. It is important to reiterate that these approaches must be integrated with the medical and surgical treatment recommended by guidelines and not replace it. An additional aspect concerns the potential heterogeneity of treatment responses based on disease phenotype. It is plausible that patients with ovarian endometrioma, superficial peritoneal endometriosis, or deep infiltrating endometriosis present different immunological and microbiological characteristics and may therefore benefit differently from nutritional and microbiome-directed strategies. Future research should therefore include stratified analyses based on the patient’s clinical phenotype. Another field of future research may concern the potential use of these interventions in combination with conservative endometriosis surgery. Currently, there is insufficient evidence to determine whether microbiome modulation or specific nutritional interventions can reduce postoperative inflammation, the risk of recurrence, or improve reproductive outcomes after complete surgical treatment.

Moving forward, the clinical management of endometriosis must pivot toward precision nutrition. By integrating multi-omics technologies, including 16S rRNA fecal sequencing, blood metabolomics, and local transcriptomics, clinicians will soon be able to profile a patient’s specific dysbiotic signature and inflammatory phenotype. This would allow for the prescription of tailored, highly bioavailable nutritional regimens that complement conventional medical therapies, rather than replacing them. Ultimately, this research framework aims to complement symptom control by supporting the reproductive microenvironment without intentional ovarian suppression; any fertility benefit requires prospective validation [121].

### 9.1. Hormonal Therapy, Menstrual Cycle, and Microbiome Interpretation

Hormonal exposure is an important potential confounder in microbiome studies of endometriosis. A recent systematic review indicates that hormonal treatments may influence reproductive-tract microbial findings, but available studies are heterogeneous and do not establish a uniform treatment-specific signature [115]. Longitudinal studies outside endometriosis show that vaginal communities can fluctuate across the menstrual cycle, with greater diversity and lower *Lactobacillus* abundance during menses in some cohorts, while combined oral contraceptive use has been associated with greater *Lactobacillus* dominance and temporal stability [47,64,117]. Future studies should record hormonal medication type/duration, menstrual-cycle phase, bleeding status, and recent antibiotic/probiotic exposure and should prefer stratified or longitudinal analyses where feasible. Table 2 summarizes the microbiome-targeted and nutritional interventions discussed above, including their main mechanisms, level of evidence, limitations, and key supporting references.

**Table 2:** Summary of microbiome-targeted and nutritional interventions in endometriosis, with evidence level and key supporting references.

Compound/Intervention	Main Mechanism	Evidence in Endometriosis	Main Limitation	Key References
N-Acetyl Cysteine (NAC)	Glutathione precursor; antioxidant/redox modulation	Observational/non-randomized human evidence plus preclinical studies	No definitive placebo-controlled efficacy or fertility data	[86,122]
Curcumin	NF-κB/COX-2-related anti-inflammatory signaling	Preclinical evidence; limited/mixed RCT data	Low bioavailability; formulation/co-therapy effects	[88,90,91,123]
Resveratrol	SIRT1-related; anti-inflammatory/anti-angiogenic	Preclinical plus limited/mixed human studies	Independent efficacy/reproductive benefit unproven	[92,93,94,95]
EGCG	VEGF/HIF-1α; anti-angiogenic/anti-fibrotic	Predominantly in vitro/animal	No validated endometriosis clinical regimen	[96,97,98,99,100,101,102]
Omega-3 PUFAs	Eicosanoid competition/pro-resolving mediators	Mechanistic evidence; heterogeneous clinical trials	Dose/endpoints vary; fertility benefit unproven	[39,103]
Vitamin D3	VDR signaling; immunomodulation	Observational and mixed interventional human evidence	Benefit may depend on deficiency	[39,104,124]
Probiotics	Strain-specific microbiome/immune modulation	Two small human trials plus preclinical work	No standardized strain/dose/duration	[41,50,65,77,117]
Prebiotics/SCFAs	Microbial fermentation; barrier/HDAC signaling	Mainly mechanistic/preclinical	No endometriosis-specific therapeutic dose	[80,81,82,83,84]
Quercetin	Antioxidant/anti-inflammatory signaling	Animal/preclinical	No endometriosis-specific human RCT	[105]
Alpha-lipoic acid	Redox cofactor/antioxidant	Open-label combination study	Independent ALA effect unknown	[106]
Vitamins C + E	Antioxidant/redox effects	Small randomized placebo-controlled trials	Short duration; no reproductive endpoint validation	[39,107]
Zinc/Selenium	Antioxidant/immune micronutrient roles	Zinc observational; selenium adjunct RCT	Routine standalone use not established	[109,110]

### 9.2. Reproductive Outcomes and Fertility-Sparing Perspectives

Endometriosis-associated infertility is multifactorial and involves altered folliculogenesis, impaired oocyte quality, reduced embryo competence, chronic peritoneal inflammation, progesterone resistance, and impaired endometrial receptivity. Nutritional and microbiome-targeted interventions are of interest because many do not intentionally suppress ovulation; however, “fertility-sparing” should not be interpreted as “fertility-enhancing.” However, current evidence linking these interventions to improved spontaneous pregnancy or ART outcomes remains limited. Future trials should therefore include reproductive endpoints such as ovulation, ovarian reserve markers, oocyte and embryo quality, implantation rate, clinical pregnancy rate, live birth rate, and miscarriage rate. Furthermore, it would be appropriate for future studies to evaluate the efficacy of these interventions using clinically relevant and shared endpoints, including patient-centered outcomes, such as improved quality of life, reduced analgesic consumption, the need for further surgery, time to conception, clinical pregnancy rate, and live birth rate. Current evidence does not establish improvement in spontaneous conception, implantation, clinical pregnancy, ART success, miscarriage, or live birth.

## 10. Study Limitations and Future Perspectives

This review has limitations. It is a narrative synthesis rather than a preregistered systematic review and did not include formal risk-of-bias assessment or meta-analysis. The underlying literature is heterogeneous in diagnostic criteria, disease phenotype, hormonal exposure, menstrual-cycle phase, dietary assessment, supplement formulation, microbiome sampling, sequencing methods, and clinical endpoints. Most microbiome studies are cross-sectional and taxonomic, limiting causal inference and direct assessment of estrobolome function; many nutraceutical data derive from cell/animal models, small trials, or combination products. Future studies should use longitudinal sampling, functional metagenomic or β-glucuronidase assays, standardized formulations, phenotype-stratified randomized trials, and shared patient-centered and reproductive endpoints, including time to conception, clinical pregnancy, and live birth. ASTRA may assist oxidative-stress hypothesis generation, but findings from generic cell lines require validation in endometriosis-relevant tissues [76].

## 11. Conclusions

In conclusion, endometriosis is no longer viewed merely as a localized pelvic disease but rather as a complex, systemic inflammatory condition driven by estrogen dominance, oxidative stress, immune dysregulation, and microbiome imbalance [125,126,127,128]. For decades, the standard of care has relied heavily on surgical excision and hormonal suppression. While these conventional therapies remain fundamental for symptom management, their inherent contraceptive nature creates a profound therapeutic paradox for the millions of women seeking to preserve or achieve pregnancy [120]. This review highlights the compelling mechanistic and preclinical evidence supporting the integration of dietary patterns, microbiome-targeted interventions, and specific bioactive compounds into the management of endometriosis [129,130,131]. By targeting the gut-pelvic axis, interventions such as probiotics and prebiotics may contribute to restoring microbial homeostasis, downregulate estrobolome-derived β-glucuronidase activity, and support intestinal barrier integrity and potentially reduce endotoxemia-related inflammatory signaling [126,127,132]. Concurrently, natural pleiotropic compounds, including N-Acetyl cysteine, curcumin, resveratrol, EGCG, pmega-3 PUFAs, and vitamin D, may act through complementary antioxidant, anti-inflammatory, anti-angiogenic, and immunomodulatory mechanisms, induce apoptosis in ectopic stromal cells, and promote anti-angiogenic and pro-resolving pathways [86,94,95,122,129,130,133]. Most importantly, in the context of women’s reproductive health, these non-suppressive interventions offer a critical advantage: they aim to rehabilitate the pelvic microenvironment rather than simply silencing ovarian function. By reducing local ROS production and modulating peritoneal cytokines, these nutritional strategies may help protect oocyte quality, improve endometrial receptivity, and support spontaneous conception and assisted reproductive technology (ART) outcomes [75,121,134]. Despite this immense potential, the transition from translational research to routine clinical practice requires overcoming substantial hurdles. The current body of clinical evidence is limited by small sample sizes, heterogeneous study designs, and the poor systemic bioavailability of many raw botanical extracts [130,131,133]. Therefore, large, multi-center, randomized, placebo-controlled trials utilizing standardized, highly bioavailable formulations (e.g., liposomes or phytosomes) are urgently required [130,133]. Looking to the future, the management of endometriosis is poised to embrace precision nutrition. Guided by multi-omics technologies, such as 16S rRNA fecal sequencing and metabolomic profiling, clinicians may become increasingly able to tailor dietary and nutraceutical prescriptions to the unique microbial and inflammatory signatures of each patient [121,127,132]. Ultimately, combining targeted nutritional and microbiome interventions with conventional medical approaches offers a truly holistic, fertility-sparing strategy. An integrative approach may therefore complement guideline-based management while addressing symptoms, relevant pathophysiological pathways, reproductive priorities, and quality of life.

## Figures and Tables

**Table 1:** Representative microbial taxa reported in studies of endometriosis and/or estrogen metabolism. Taxonomic associations are summarized separately from functional estrobolome evidence; neither constitutes a validated causal or diagnostic signature.

Site/Taxon	Reported Association or Functional Relevance	Evidence/Interpretation	References
**Gut:** ***Bacteroides*** **spp.,** ***Clostridium*** **spp.,** ***Ruminococcus*** **spp.**	Some members encode β-glucuronidases capable of estrogen deconjugation.	Functional capacity is gene/strain dependent; abundance alone does not establish estrobolome activity or endometriosis causality.	[35,46,47]
**Gut/reproductive tract:** ***Lactobacillus*** **spp.**	Differences in relative abundance have been reported in some endometriosis cohorts.	Associations are heterogeneous; species/strain resolution is important.	[20,49,63,66,67]
**Vaginal:** ***L. crispatus*** **,** ***L. gasseri*** **,** ***L. iners*** **,** ***L. jensenii***	Representative dominant species in established vaginal community states.	Not an endometriosis-specific diagnostic signature.	[64,65]
**Reproductive tract:** ***Escherichia/Shigella*** **,** ***Streptococcus*** **,** ***Ureaplasma***	Altered relative abundance reported in some cohorts.	Findings vary by site/study; no validated microbial biomarker.	[49,66]

## Data Availability

No new data were created or analyzed in this study. Data sharing is not applicable to this article.
